# Efficacy and Safety of Vagus Nerve Stimulation for Hospitalized COVID-19 Patients: A Systematic Review and Methodological Evaluation of Randomized Controlled Trials

**DOI:** 10.3390/medicina62040649

**Published:** 2026-03-28

**Authors:** Adrian Balan, Giles Graham, Herban Sorin, Marius Marcu, Nini Gheorghe, Mara Gabriela, Andreea-Roxana Florescu, Alina-Mirela Popa, Ana Lascu, Cristian Ion Mot, Stefan Mihaicuta, Stefan Marian Frent

**Affiliations:** 1 Center for Research and Innovation in Precision Medicine of Respiratory Diseases, Department of Pulmonology, University of Medicine and Pharmacy Timisoara, 300041 Timisoara, Romania; adrian.balan.umfvbt@gmail.com (A.B.); roxana95andreea@gmail.com (A.-R.F.); alina-mirela.popa@umft.ro (A.-M.P.); stefan.mihaicuta@umft.ro (S.M.); frentz.stefan@umft.ro (S.M.F.); 2 Europe to Europe, 78 Providence Park, Southampton SO16 7QW, UK; drgrahamgiles@yahoo.co.uk; 3 Royal Society of Medicine, 1 Wimpole Street, London W1G OAE, UK; 4 Department of Civil Engineering and Installations, Polytechnic University of Timisoara, 300006 Timisoara, Romania; sorin.herban@upt.ro; 5 Department of Computer and Software Engineering, Polytechnic University of Timisoara, 300006 Timisoara, Romania; marius.marcu@cs.upt.ro; 6 Faculty of Medicine, Vasile Goldis Western University of Arad, 310414 Arad, Romania; nini.gheorghe@uvvg.ro (N.G.); mara.gabriela@uvvg.ro (M.G.); 7 Multidisciplinary Doctoral School, Vasile Goldis Western University of Arad, 310414 Arad, Romania; 8 Department of Doctoral Studies, University of Medicine and Pharmacy Timisoara, Eftimie Murgu Square No. 2, 300041 Timisoara, Romania; 9 Institute of Pulmonology “Marius Nasta” Bucharest, 050159 Bucharest, Romania; 10 Department of Functional Sciences, Discipline of Pathophysiology, Centre for Translational Research and Systems Medicine, University of Medicine and Pharmacy Timisoara, Eftimie Murgu Square No. 2, 300041 Timisoara, Romania; 11 Institute for Cardiovascular Diseases of Timisoara, Clinic for Cardiovascular Surgery, 300310 Timisoara, Romania; 12 Otorhinolaryngology Department, Municipal Emergency Hospital Timisoara, 300041 Timisoara, Romania; ion.mot@umft.ro; 13 Department of Surgery, University of Medicine and Pharmacy Timisoara, Eftimie Murgu Square No. 2, 300041 Timisoara, Romania

**Keywords:** vagus nerve stimulation, COVID-19, SARS-CoV-2, systematic review, randomized controlled trials, risk of bias, cholinergic anti-inflammatory pathway, cytokine storm, neuromodulation, AI tools

## Abstract

*Background and Objectives*: Coronavirus disease 2019 (COVID-19) is characterized by excessive inflammatory responses, including the so-called cytokine storm, which contributes substantially to morbidity and mortality in hospitalized patients. The vagus nerve, through the cholinergic anti-inflammatory pathway, represents a theoretically attractive therapeutic target for modulating systemic inflammation. Vagus nerve stimulation (VNS) has emerged as a potential adjunctive treatment for COVID-19, with several randomized controlled trials (RCTs) investigating its efficacy on inflammatory biomarkers and clinical outcomes. The quality of this evidence base has not been rigorously evaluated. This systematic review critically appraises all available RCT evidence for VNS in hospitalized COVID-19 patients. *Materials and Methods*: We systematically searched PubMed, Scopus, Cochrane (CENTRAL), and Web of Science from database inception to January 2026, for RCTs evaluating any form of VNS (invasive, non-invasive, cervical, or auricular) in hospitalized patients with confirmed acute COVID-19. Two reviewers independently screened titles, abstracts, and full texts according to pre-specified eligibility criteria. Risk of bias was assessed using the Cochrane Risk of Bias 2 (RoB 2) tool, with assessments initially performed using multiple artificial intelligence tools and subsequently validated by the authors in accordance with PRISMA 2020 guidelines. Given substantial heterogeneity and high risk of bias, narrative synthesis was performed rather than meta-analysis. Also, GRADE assessment was performed. *Results*: From 437 records identified, six RCTs comprising 221 patients met the inclusion criteria. Five trials (83%) were rated as high risk of bias, primarily due to inadequate blinding, substantial baseline imbalances, significant missing data and extensive multiple testing without statistical correction. The single double-blind trial with a credible sham control (Rangon et al.) found null results across all outcomes, including clinical progression, ICU transfer, and mortality, while the five “high” risk-of-bias trials generally reported positive findings on various inflammatory markers and clinical outcomes. One trial (Corrêa et al.) measured heart rate variability as a direct indicator of vagal activation and found no change despite claiming anti-inflammatory effects, contradicting the proposed mechanism of action. Significant cognitive findings from an interim analysis (Uehara et al., *n* = 21) disappeared in the larger completed trial (Corrêa et al., *n* = 52), providing empirical demonstration of false positive findings in small, underpowered studies. *Conclusions*: Currently available evidence supporting the use of VNS for acute COVID-19 remains scarce; however, the physiological rationale remains sound, although the absence of reliable target engagement markers in the included studies limits confidence in this treatment method. Large-scale, double-blind, sham-controlled trials are required before VNS can be firmly recommended for COVID-19 management.

## 1. Introduction

### 1.1. COVID-19 and the Systemic Inflammatory Response

The coronavirus disease 2019 (COVID-19) pandemic, caused by (SARS-CoV-2), has resulted in unprecedented global morbidity and mortality, with over 700 million confirmed cases and nearly 7 million deaths reported worldwide as of late 2023. While most SARS-CoV-2 infections result in mild or asymptomatic illness, a substantial proportion of patients (particularly those with advanced age, obesity, diabetes mellitus, cardiovascular disease, and other comorbidities develop severe disease characterized by progressive respiratory failure, acute respiratory distress syndrome (ARDS), multi-organ dysfunction syndrome, and death [1,2].

Central to the pathophysiology of severe COVID-19 is an exaggerated or dysregulated inflammatory response, usually termed the “cytokine storm” or cytokine release syndrome. This hyperinflammatory state is characterized by excessive production of pro-inflammatory cytokines and chemokines (IL-6, IL-1β, TNF-α, IFN-γ, IL-18, MIP-1α, VEGF). The cytokine storm contributes directly to alveolar epithelial damage, endothelial dysfunction, vascular leak syndrome, disseminated intravascular coagulation, and the hyperinflammatory state that underlies much of COVID-19-associated mortality. Elevated serum levels of C-reactive protein (CRP), ferritin, D-dimer, procalcitonin, and lactate dehydrogenase have been consistently and independently associated with disease severity and poor prognosis, serving as important prognostic biomarkers in clinical practice [3,4,5].

The recognition that hyperinflammation drives COVID-19 severity has led to intensive investigation of anti-inflammatory therapeutic strategies. Corticosteroids, particularly dexamethasone, have demonstrated mortality benefit in patients requiring supplemental oxygen and are now established as standard of care [6]. Interleukin-6 receptor antagonists (tocilizumab, sarilumab) and Janus kinase inhibitors (baricitinib) have shown benefit in selected populations, though effects are modest and patient selection remains challenging [7,8]. Despite these advances, mortality in severe COVID-19 remains substantial, and additional therapeutic approaches targeting the inflammatory cascade continue to be explored.

Beyond the acute inflammatory response, dysregulation of the cholinergic system has been implicated in COVID-19 pathogenesis. SARS-CoV-2 may directly or indirectly affect cholinergic signaling, potentially contributing to the multi-organ manifestations of the disease, including cardiovascular complications, gastrointestinal symptoms, and neurological sequelae [9]. This observation has led to interest in therapeutic approaches that modulate cholinergic pathways, including vagus nerve stimulation.

### 1.2. The Vagus Nerve and Neuroimmune Communication

The vagus nerve, the tenth cranial nerve and principal component of the parasympathetic nervous system, plays a critical role in the bidirectional communication between the central nervous system and peripheral organs, including modulation of the immune response. Comprising approximately 80% afferent (sensory) fibers and 20% efferent (motor) fibers, the vagus nerve innervates the heart, lungs, gastrointestinal tract, liver, spleen, and other visceral organs, enabling real-time neural monitoring and regulation of peripheral physiological processes [3,10].

In a landmark discovery published in Nature in 2000, Borovikova and colleagues demonstrated that electrical stimulation of the vagus nerve attenuates the systemic inflammatory response to endotoxin (lipopolysaccharide) in rodents, significantly reducing serum TNF-α levels and preventing the development of endotoxic shock [11]. This observation established the concept of neural control of inflammation and led to the subsequent identification of the “inflammatory reflex” by Kevin Tracey and colleagues [12,13]. The inflammatory reflex describes a neural circuit whereby the nervous system reflexively monitors and modulates the peripheral inflammatory response through vagal signaling, similar to other homeostatic reflexes controlling heart rate, blood pressure, and respiration.

The efferent arm of this reflex, termed the cholinergic anti-inflammatory pathway (CAP), operates through vagal release of acetylcholine binding to alpha-7 nicotinic acetylcholine receptors (α7nAChR) on tissue macrophages, dendritic cells, and other immune cells in lymphoid organs. This activation inhibits the nuclear factor kappa-B (NF-κB) signaling cascade, suppressing pro-inflammatory cytokines (TNF-α, IL-1β, IL-6, HMGB1) without impairing anti-inflammatory cytokine production or host defense. The CAP has been extensively validated in preclinical models of endotoxemia, sepsis, ischemia-reperfusion injury, hemorrhagic shock, and autoimmune conditions, with surgical vagotomy or pharmacological vagal blockade consistently exacerbating inflammation—confirming the tonic anti-inflammatory role of vagal activity [5,13,14].

However, neuroimmune pathways that are consistently demonstrable in controlled preclinical settings frequently exhibit attenuated or heterogeneous effects in human disease. This occurs where inflammatory states are more complex, treatment contexts are more variable, and engagement of physiological pathways is more difficult to verify—raising the translational challenge that warrants careful evaluation of the clinical evidence.

The auricular branch of the vagus nerve (ABVN, Arnold’s nerve) provides percutaneous access to vagal afferents through the external ear, most consistently via the cymba conchae. Functional MRI studies confirm that auricular stimulation activates brainstem vagal nuclei, including the nucleus tractus solitarius and locus coeruleus. Alternatively, the cervical vagus trunk can be accessed non-invasively via transcutaneous stimulation of the lateral neck [15,16,17].

### 1.3. Vagus Nerve Stimulation as Therapeutic Intervention

Vagus nerve stimulation (VNS) has been employed therapeutically for drug-resistant epilepsy since 1997 and treatment-resistant depression since 2005, using surgically implanted devices [18,19]. The development of non-invasive transcutaneous VNS (tVNS) techniques has substantially expanded potential clinical applications without surgical risks, reducing costs, and enabling broader accessibility for research and clinical use [20].

Non-invasive VNS has been investigated in diverse conditions with varying degrees of evidence supporting efficacy. In migraine prevention and acute treatment, a tVNS device has received regulatory approval based on randomized controlled trial evidence [21]. In rheumatoid arthritis, VNS has demonstrated reductions in disease activity and inflammatory markers in pilot studies [22]. Studies in inflammatory bowel disease (Crohn’s disease), postoperative ileus, atrial fibrillation, and heart failure have shown variable results, with some evidence of anti-inflammatory and clinical benefits but substantial heterogeneity across studies. More recently, scoping reviews have systematically mapped the expanding clinical applications of tVNS across neurology, cardiology, psychiatry, gastroenterology, and immunology [23,24,25].

However, evidence for anti-inflammatory effects of VNS in humans remains inconsistent. Recent meta-analyses have reported no consistent anti-inflammatory effects across various conditions, significant heterogeneity in studies examining VNS-mediated inflammation modulation, and mixed results for auricular stimulation on inflammatory parameters [26,27,28].

Despite emerging RCT evidence examining VNS in acute COVID-19, this literature has not been subjected to a rigorous systematic review. Existing trials differ substantially in stimulation modality, patient population or outcome selection, and a prior meta-analysis (the only quantitative synthesis to date) contains significant methodological limitations that call its conclusions into question. The absence of a bias-informed evaluation means that current understanding of VNS efficacy in COVID-19 rests on an inadequately scrutinized evidence base, justifying the present endeavor.

### 1.4. Study Objectives

The objectives of this systematic review are:

(1) to identify and describe all randomized controlled trials evaluating vagus nerve stimulation in hospitalized patients with acute COVID-19;
(2) to assess methodological quality using the Cochrane Risk of Bias 2 (RoB 2) tool;
(3) to synthesize evidence on the efficacy of VNS on clinical outcomes, inflammatory biomarkers, and adverse events using a comprehensive, holistic approach;
(4) to provide recommendations for clinical practice and future research.

## 2. Materials and Methods

This systematic review was designed, conducted, and reported in strict accordance with the Preferred Reporting Items for Systematic Reviews and Meta-Analyses (PRISMA) 2020 statement [29]. The review protocol was developed following detailed recommendations from the Cochrane Handbook for Systematic Reviews of Interventions, which represents the current gold standard for systematic review methodology and provides comprehensive guidance on all aspects of systematic review conduct [30]. The review protocol was prospectively registered in the International Prospective Register of Systematic Reviews (PROSPERO). The registration number is CRD420261287286. The PRISMA checklist can be found in the Supplementary Materials. All methodological decisions (including eligibility criteria using the PICOS framework, search strategy, screening procedures, data extraction items, risk of bias assessment approach using RoB 2, and planned synthesis methods) were documented a priori in the review protocol before data synthesis was undertaken, which will be available at the publishing date. Any possible change or protocol deviation was discussed in the review team and reported accordingly. The review was conducted in accordance with the protocol; no amendments or deviations were required. The review originated as part of a comprehensive literature evaluation to contextualize our own clinical research findings and evolved into a formal systematic review as the analysis progressed.

### 2.1. Eligibility Criteria

Population: Adult patients (≥18 years of age) with confirmed COVID-19 (laboratory-confirmed SARS-CoV-2 infection by reverse transcription polymerase chain reaction [RT-PCR] or antigen testing) requiring hospitalization for acute illness.

Intervention: Any form of vagus nerve stimulation, including invasive (surgically implanted) VNS, non-invasive transcutaneous cervical VNS (tcVNS), transcutaneous auricular VNS (taVNS), and percutaneous auricular VNS (paVNS). All stimulation parameters, frequencies, intensities, and treatment durations were eligible for inclusion.

Comparator: Standard of care (as defined by the individual study protocol and institutional guidelines) with or without sham stimulation. Sham stimulation could include inactive devices, stimulation of non-vagal auricular regions, or other placebo procedures.

Outcomes: Studies were required to report at least one of the following outcome categories: (1) inflammatory biomarkers (including but not limited to CRP, IL-6, TNF-α, IL-1β, IL-10, ferritin, procalcitonin, D-dimer); (2) clinical outcomes (mortality, intensive care unit admission, mechanical ventilation, non-invasive ventilation, length of hospital stay, clinical progression scales); or (3) safety outcomes (adverse events, tolerability).

Study design: Randomized controlled trials only.

Exclusion criteria: Studies were excluded for the following reasons: (1) non-randomized study designs (2) case reports or case series; (3) literature review articles (4) preclinical studies (animal models, in vitro experiments); (5) studies not published in English (searches were restricted to English-language publications due to feasibility constraints, as the absence of certified translators; this restriction is acknowledged as a potential source of language bias); (6) conference abstracts with insufficient methodological detail (7) studies conducted exclusively in healthy volunteers without COVID-19; (8) studies focused on post-acute sequelae of SARS-CoV-2 infection (PASC), long-COVID syndrome, or post-COVID conditions rather than acute illness.

### 2.2. Information Sources and Search Strategy

We systematically searched four electronic databases from inception to January 2026 (last search date 20 January 2026): PubMed (including MEDLINE), Scopus, Cochrane Central Register of Controlled Trials (CENTRAL), and Web of Science Core Collection. No date restrictions were applied. The search strategy combined controlled vocabulary terms (Medical Subject Headings [MeSH] in PubMed, Emtree terms in Scopus where applicable) with free-text keywords to maximize sensitivity.

PubMed search strategy: (“COVID-19”[MeSH] OR “COVID-19” OR “SARS-CoV-2”[MeSH] OR “SARS-CoV-2” OR “Coronavirus”[MeSH] OR “Coronavirus”) AND (“Vagus Nerve”[MeSH] OR “Vagus Nerve” OR “Vagus Nerve Stimulation”[MeSH] OR “Vagus Nerve Stimulation” OR “transcutaneous vagus nerve stimulation” OR “vagal nerve” OR “vagal nerve stimulation”)

Scopus, Cochrane CENTRAL, and Web of Science search strategy: (“COVID-19” OR “SARS-CoV-2”) AND “vagus nerve stimulation.”

Additional search methods included manual review of reference lists from included studies and relevant systematic reviews to identify potentially eligible studies not captured by database searches. We also searched ClinicalTrials.gov and the International Clinical Trials Registry Platform (ICTRP) for registered trials and compared these against published studies to assess for potential publication bias and identify unpublished trials. To enhance the completeness of the literature search, citation chasing was conducted using “ResearchRabbit” (2025, Version 2025-11-18, last accessed January 2026). For each included study, reference lists were reviewed (backward citation tracking), and citing articles were identified (forward citation tracking). This process was undertaken to capture additional relevant publications not identified through database searching alone and to verify the robustness of the search strategy.

No study-type filters were applied at the search stage in any database. No language restriction was applied during searching; restriction to English-language publications was applied as an eligibility criterion during screening and is acknowledged as a limitation.

### 2.3. Study Selection Process

**Figure 1:**
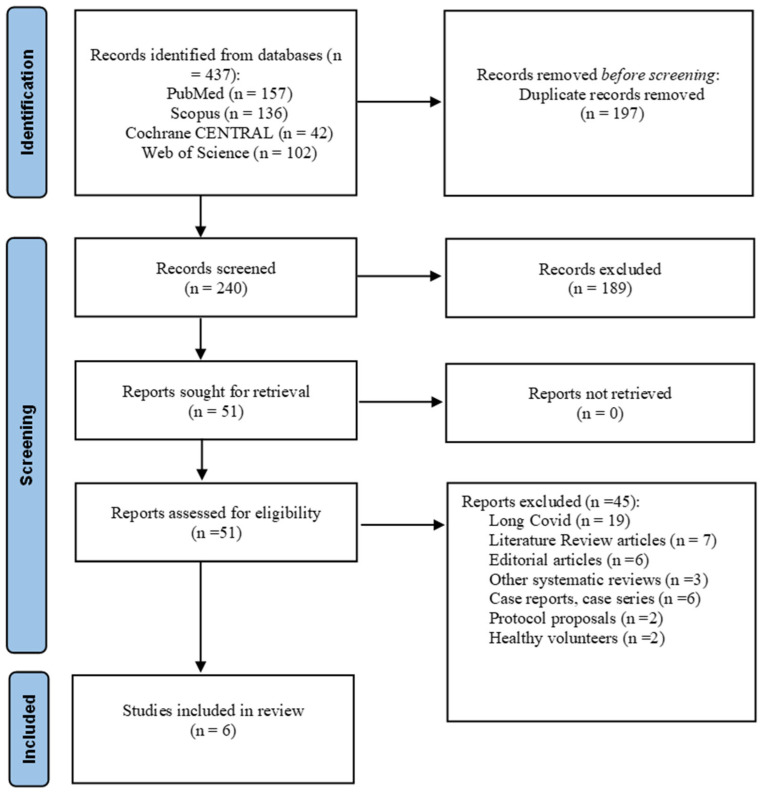
PRISMA 2020 Flow Diagram.

All records identified from database searches were manually compared, and duplicate records were removed. Two of the review team members independently screened titles and abstracts to identify potentially relevant studies. Full-text articles were retrieved for all records that appeared to meet eligibility criteria or for which eligibility could not be determined from the title and abstract alone. The same two reviewers independently assessed full-text articles against the pre-specified inclusion and exclusion criteria. Disagreements at any stage were resolved through discussion and consensus; if consensus could not be reached, a third reviewer adjudicated. The study selection process is presented in the PRISMA flow diagram (Figure 1).

### 2.4. Data Extraction

Data was extracted manually by the team members. Discrepancies were resolved by discussion and reference to the original publication. The following data items were extracted: study identifiers: first author, year of publication, country, journal, trial registration number, funding sources; study design characteristics: randomization method, allocation concealment, blinding (participants, personnel, outcome assessors), control type (standard of care, sham stimulation); participant characteristics: sample size (randomized and analyzed), age, sex distribution, disease severity at baseline (oxygen requirements, inflammatory markers), relevant comorbidities; intervention details: VNS type and device, stimulation parameters (frequency, intensity, pulse width, duty cycle), treatment duration, number of sessions; comparator details: standard of care components, sham procedure description if applicable; outcomes: all reported efficacy outcomes with effect estimates and measures of precision, adverse events; results: point estimates, measures of variability (standard deviation, standard error, interquartile range, confidence intervals), sample sizes, *p*-values. Baseline characteristics were extracted in detail to evaluate the balance between treatment groups and identify potential confounding. When multiple publications reported on the same study population or trial registration, data were extracted from the most comprehensive report, with Supplementary Information obtained from additional publications as needed.

### 2.5. Risk of Bias Assessment

Risk of bias was assessed using the Cochrane Risk of Bias 2 (RoB 2) tool for randomized trials [30].

A novel artificial intelligence-assisted approach was employed for the initial phase of risk of bias assessment to minimize subjective bias and ensure comprehensive consideration of methodological features. Each included study was independently analyzed using four distinct AI systems: GPT-5.2 version (OpenAI, San Francisco, California, USA), Claude Sonnet 4.5 and Claude Opus 4.5 (Anthropic, 548 Market Street, San Francisco, CA 94104), and Microsoft Copilot (based on GPT-5) (One Microsoft Way, Redmond, WA 98052). Identical verbatim prompts were administered to all four AI following full-text document upload for each included study, ensuring complete standardization of inputs across platforms. Each AI system generated its assessments independently without access to the outputs of the other systems, preserving mutual blinding throughout the initial assessment phase. The resulting domain-level judgments and supporting rationales were subsequently reconciled through two iterative synthesis rounds using Claude Opus 4.5, which identified discordances across tools and produced consolidated assessments for human review. Inter-AI agreement was not formally quantified using a statistical coefficient, which represents an acknowledged limitation of this procedure.

The assessments from each AI tool were compiled and structured by the RoB 2 domain for each study. These preliminary assessments were then synthesized through two iterative rounds of review using Claude Opus 4.5, which reconciled discrepancies across tools, identified areas of uncertainty, and produced consolidated domain-level judgments with detailed supporting rationales. Finally, the AI-generated assessments were critically reviewed and validated by the human authors with constant reference to the RoB 2 guidance document and PRISMA guidelines. Two of the authors independently performed the full RoB 2 assessment under constant supervision from the team, and then viewed the AI output. Between the human reviewers, the inter-rater agreement was calculated as a percentage, given that the small number of included trials (*n* = 6) renders the kappa coefficient statistically unreliable and inappropriate for this context. Agreement across individual RoB 2 domains reached 96.66%, and overall risk-of-bias judgements were in full agreement between reviewers (100%). Discrepancies were resolved through discussion and consensus with the wider review team. The AI results were used as comprehensive drafts, and all the final considerations were made by human reviewers. The authors verified the accuracy of AI-extracted information against the original publications, ensured correct application of RoB 2 signaling questions, and made final determinations on domain judgments where AI assessments were ambiguous or inconsistent, although the agreement between human assessment and AI output was substantial (the authors acknowledge that formal inter-rater agreement statistics were not calculated, and this could be considered a limitation). Any possible discrepancies were resolved through discussion and consensus among the reviewing team. The authors acknowledge that the incorporation of AI tools into RoB 2 assessment is not yet formally standardized in systematic review methodology; this multi-layered approach was designed as a supplementary measure to reduce the risk of oversight and to combine the comprehensiveness and consistency of AI-assisted analysis with the clinical and methodological expertise of human reviewers.

### 2.6. Data Synthesis

We initially planned to conduct a random-effects meta-analysis if sufficient clinical and methodological homogeneity existed among included studies. However, after data extraction and risk of bias assessment, we determined that quantitative synthesis would be inappropriate for the following reasons: Clinical heterogeneity: Studies employed substantially different VNS modalities (cervical vs. auricular), devices (gammaCore, AuriStim, custom devices), stimulation parameters (frequency ranging from 1 to 25 Hz, varying intensities and duty cycles), and treatment durations (single session to 14+ days). Methodological heterogeneity: Control conditions ranged from standard of care alone to sham stimulation, with substantial variation in the credibility of blinding procedures. Outcome heterogeneity: Different inflammatory markers, measurement timepoints, and clinical outcome definitions precluded meaningful pooling. High risk of bias: Most included trials were rated as high risk of bias; pooling such studies may compound rather than mitigate bias, producing misleading precision around biased estimates [30,31].

Given these considerations, data were synthesized narratively with emphasis on risk of bias findings and their implications for the interpretation of efficacy signals. Results are presented descriptively by outcome category, and the pattern of findings is examined in relation to methodological quality, consistent with Cochrane guidance on synthesis without meta-analysis (SWiM) [32].

## 3. Results

### 3.1. Study Selection

The electronic database searches identified 437 records: PubMed (*n* = 157), Scopus (*n* = 136), Cochrane CENTRAL (*n* = 42), and Web of Science (*n* = 102). After the removal of 197 duplicate records, 240 unique records remained for title and abstract screening. Of these, 189 records were excluded as clearly ineligible based on title and abstract review (primarily review articles, commentaries, preclinical studies, and studies in unrelated conditions). Fifty-one full-text articles were retrieved and assessed for eligibility against the pre-specified inclusion and exclusion criteria. Forty-five full-text articles were excluded for the following reasons: non-randomized study design, review article, commentary, or editorial, study of long-COVID syndrome or post-acute sequelae, study in healthy volunteers without COVID-19, protocol publication without results, and insufficient methodological detail to permit assessment. No conference abstracts meeting the eligibility criteria were identified during the systematic search. Six randomized controlled trials met all eligibility criteria and were included in the qualitative synthesis [33,34,35,36,37,38] (Figure 1).

### 3.2. Study Characteristics

The six included randomized controlled trials comprised a total of 221 participants (range: 10–97 per study) and were conducted between 2020 and 2022 in four countries: Spain, France, Austria, and Brazil. Study characteristics are summarized in Table 1.

Tornero and colleagues conducted the largest trial (SAVIOR-I), a randomized, open-label study comparing non-invasive cervical VNS using the gammaCore Sapphire device (electroCore Inc., Rockaway, NJ, USA) plus standard of care versus standard of care alone in 97 evaluable hospitalized COVID-19 patients (47 VNS, 50 control) in Spain. Patients received bilateral cervical stimulation (two consecutive 2 min doses, three times daily) until hospital discharge or up to 15 days. The primary outcomes were changes in inflammatory biomarkers (CRP, procalcitonin, D-dimer, ferritin, cytokines) and clinical respiratory outcomes.

Rangon and colleagues conducted a multicenter, randomized, double-blind, sham-controlled pilot study (SOS COVID-19) evaluating auricular neuromodulation using semi-permanent acupuncture needles placed at vagally innervated auricular acupoints in 29 hospitalized COVID-19 patients (14 active, 15 sham) in France [38]. The sham procedure consisted of pressure application using empty needle applicators at the same acupoints, with both groups having their ears covered by opaque bandages to maintain blinding. The primary outcome was changed in the 7-point WHO Clinical Progression Scale on Day 14.

Two trials from the same Austrian research group evaluated percutaneous auricular VNS using the AuriStim device (Multisana GmbH, Murnau am Staffelsee, Germany): Seitz et al. (2022) reported inflammatory marker outcomes in 10 patients (5 VNS, 5 control) [36], while Seitz et al. (2023) reported clinical outcomes in 12 patients (6 VNS, 6 control) [37]. Both were open-label trials without a sham control. The AuriStim device delivers percutaneous stimulation via miniature needle electrodes inserted into vagally innervated regions of the auricle, with an intermittent duty cycle (3 h on, 3 h off). The potential overlap between these two publications, whether they represent partially or entirely overlapping patient cohorts with different outcome reporting, could not be definitively determined from the published information.

Two publications from a Brazilian research group reported on transcutaneous auricular VNS in hospitalized COVID-19 patients: Uehara et al. (2022) reported cognitive and inflammatory outcomes in 21 patients [35], while Corrêa et al. (2022) reported inflammatory, autonomic (heart rate variability), and cognitive outcomes in 52 patients [34]. These publications share the same trial registry number (RBR-399t4g5), overlapping authorship, identical institutional affiliation, and similar outcome measures. The Uehara publication (*n* = 21) appears to represent an interim analysis of the larger Corrêa trial (*n* = 52), meaning findings from these publications are not independent. Both were open-label trials without a sham control.

### 3.3. Risk of Bias Assessment

Risk of bias assessments are summarized in Table 2, with detailed domain-level judgments and supporting rationales presented in Table 3. Overall, five of six trials (83%) were rated as “high” risk of bias, and one trial (Rangon et al.) [38] was rated as having “some concerns.” No trial achieved “low” risk of bias across all domains. The predominant sources of bias were lack of blinding (Domain 2), baseline imbalances (Domain 1), and multiple testing without correction (Domain 5).

### 3.4. Domain 1: Bias Arising from the Randomization Process

Substantial baseline imbalances were identified in several trials that may have systematically favored the detection of treatment effects. In Seitz et al. (2022) [36], baseline inflammatory markers were markedly higher in the VNS group than in controls despite randomization: TNF-α was approximately 3-fold higher (mean 19.3 vs. 6.3 pg/mL), and IL-6 was 2.6-fold higher (mean 341.4 vs. 129.0 pg/mL).

In Corrêa et al. (2022) [34], baseline IL-6 was 34% higher, and CRP was 22% higher in the control group compared to the VNS group, potentially underestimating treatment effects (opposite direction of bias) or representing chance imbalance. These baseline differences were not adjusted in the primary analysis. The Rangon trial [38] showed a 10-year age difference between groups (median 75.5 vs. 65 years) with substantial gender imbalance (79% vs. 47% male), potentially confounding clinical outcomes given the well-established associations between advanced age, male sex, and COVID-19 severity and mortality. The Tornero trial [33] reported significantly different mean ages between groups (55.5 vs. 61.3 years, *p* = 0.022) and higher disease severity in the VNS group at baseline (15% vs. 4% severe cases).

### 3.5. Domain 2: Bias Due to Deviations from Intended Interventions

Five of six trials (83%) were open-label without a sham control, representing a fundamental methodological limitation. Only the Rangon trial employed double-blinding with a credible sham procedure (pressure application with an empty needle applicator, ears covered by opaque bandages).

In Seitz et al. (2023) [37], the implausibly large effect sizes for clinical outcomes (0% intubation in VNS versus 50% in controls, 0% ECMO requirement versus 67% in controls) may reflect confounding by indication, expectation bias, or other systematic bias rather than true treatment effects of this magnitude, considering the neuromodulation effects evaluated in other inflammatory conditions.

A particularly concerning finding emerged in Corrêa et al. (2022) [34], which reported a striking co-intervention imbalance: 100% of patients in the VNS group received gastrointestinal drugs compared to only 39% of controls (*p* = 0.001). This represents a systematic deviation from the intended intervention that was neither explained nor adjusted for in the analysis.

### 3.6. Domain 3: Bias Due to Missing Outcome Data

The Tornero (SAVIOR-I) trial [33] had substantial missing data, with 13 of 110 randomized participants (12%) excluded from analysis due to missing baseline data, and 40–50% missing laboratory values at various follow-up timepoints. The authors acknowledged that “consistent data collection was challenging in the environment of the pandemic.” Importantly, no sensitivity analyses or multiple imputation methods were employed to assess whether missing data may have biased results. Given that missingness was likely related to disease severity (sicker patients may have been less likely to have blood draws or may have died before follow-up measurements), this represents potentially informative (non-random) missingness that could systematically bias the results.

Corrêa et al. (2022) [34] reported differential missingness for heart rate variability data: 7.7% of VNS patients versus 19.2% of control patients were excluded from HRV analysis. This discrepancy, with more missing data in the control group, was not investigated or adjusted for.

### 3.7. Domain 4: Bias in Measurement of Outcomes

For objective laboratory outcomes (inflammatory biomarker concentrations), outcome measurement procedures were generally appropriate and unlikely to be influenced by knowledge of treatment assignment, as laboratory analyses are performed by automated equipment without subjective interpretation. However, for subjective outcomes and clinical decision-based outcomes, the open-label design introduced substantial risk of measurement and ascertainment bias.

In Uehara et al. (2022) [35], cognitive outcomes were assessed by unblinded assessors using neuropsychological tests that require examiner judgment in administration and scoring, introducing expectation bias. In Seitz et al. (2023) [37], clinical decisions regarding intubation timing and ECMO initiation were made by clinicians with knowledge of treatment assignment.

### 3.8. Domain 5: Bias in Selection of Reported Results

Multiple testing without statistical correction was universal across trials, substantially inflating the family-wise false positive rate. Corrêa et al. (2022) [34] assessed approximately 30 distinct outcomes across multiple timepoints and analytic approaches without any adjustment for multiplicity.

Seitz et al. (2022) [36] measured eight distinct inflammatory markers at up to 20 timepoints per patient, with between-group comparisons at each timepoint, generating hundreds of statistical tests without multiplicity correction. Tornero et al. explicitly stated that “there were no adjustments for multiple comparisons” despite testing numerous inflammatory markers and clinical outcomes. The trial was also industry-funded (electroCore), introducing potential conflicts of interest in outcome selection and reporting.

The relationship between the Uehara [35] and Corrêa [34] publications raises additional concerns about selective reporting. Significant cognitive findings reported in Uehara (memory performance *p* = 0.01, attention *p* = 0.04 in *n* = 21) were no longer statistically significant in the larger Corrêa sample (*n* = 52).

### 3.9. JADAD Scale Assessment

As a supplementary assessment of methodological quality, all six included RCTs were evaluated using the JADAD scale, a validated five-point instrument assessing randomization, double-blinding, and reporting of withdrawals [39,40]. The assessment was performed by the same two reviewers; inter-rater agreement was calculated as a percentage given the small number of included studies, yielding a 100% agreement rate. Results were verified through discussions and consensus with the rest of the team. Scores ranged from 1 to 5 out of a possible maximum of 5. The single adequately blinded trial (Rangon et al. [38]) achieved the maximum score of 5/5, consistent with its double-blind, sham-controlled design and complete reporting of participant flow. Tornero et al. (SAVIOR-I) [33] and Corrêa et al. [34] each scored 3/5, reflecting adequate randomization and withdrawal reporting but penalized for the absence of blinding. Seitz et al. 2023 [37] and Uehara et al. [35] each scored 2/5, while Seitz et al. 2022 [36] received the lowest score of 1/5, attributable to an insufficiently described randomization method, absence of blinding, and failure to report withdrawals. The full assessment chart is presented in Table 4.

### 3.10. PEDro Scale Assessment

We performed a complementary assessment using the PEDro scale, an eleven-item instrument evaluating both internal validity and statistical reporting quality of clinical trials [41,42]. The same two reviewers assessed the trials independently, achieving a 100% inter-rater agreement rate. The assessment was verified in discussion with the authorship team. Item 1 refers to the specification of eligibility criteria and is not included in the total score. Items 2 through 11 are each scored dichotomously. Scores ranged from 4 to 9 out of a maximum of 10. Rangon et al. achieved a score of 9/10, satisfying all criteria except for therapist blinding, confirming its status as the methodologically superior trial in this evidence base. Tornero et al. (SAVIOR-I) scored 5/10, satisfying criteria for random allocation, adequate follow-up, between-group statistical comparisons, and reporting of point measures and variability, but failing all blinding and allocation concealment criteria and the intention-to-treat requirement. The remaining four trials (Seitz et al. 2022 [36], Seitz et al. 2023 [37], Corrêa et al. and Uehara et al.) each scored 4/10, satisfying only the criteria for random allocation, follow-up completeness (where applicable), between-group statistical comparisons, and reporting of point measures with variability. None of these four trials satisfied any blinding criterion at the level of subjects, therapists, or assessors, nor did any report an intention-to-treat analysis or adequate allocation concealment. Collectively, PEDro scores below 6 (the conventional threshold for good methodological quality) in five of six included trials are consistent with the high risk of bias identified through Cochrane RoB 2 and JADAD Scale assessment results. The detailed findings are presented in Table 5.

### 3.11. GRADE Assessment of Evidence Certainty

The certainty of evidence was assessed using the Grading of Recommendations Assessment, Development and Evaluation (GRADE) framework for each patient-important outcome [43,44], across five domains [43,45,46,47,48,49]. This assessment was performed independently by the same two reviewers who performed the RoB 2 assessment and verified through iterative discussion and consensus among the full authorship team. No factors warranting upgrading were identified. The GRADE Evidence Profile can be checked in Table 6, and the Summary of Findings is presented in Table 7.

For inflammatory biomarkers (CRP, IL-6, TNF-α), the certainty of evidence was rated very low, downgraded for very serious risk of bias (83% of contributing trials at high risk of bias due to open-label designs, massive baseline imbalances in Seitz 2022 [36] creating regression-to-the-mean artifacts, 40–50% missing data in Tornero, and universal absence of multiplicity correction), very serious inconsistency (contradictory findings across trials, with positive CRP results in Tornero nullified by the larger Corrêa trial showing no between-group differences after accounting for multiple comparisons), and very serious imprecision (total N = 171 across four trials, ranging from 10 to 97 participants per study, with wide confidence intervals) [33,34,35,36]. Selective reporting was suspected based on the Uehara–Corrêa interim publication pattern and industry funding [33,34,35].

For clinical outcomes (mortality, ICU admission, mechanical ventilation, ECMO), the certainty was rated very low, downgraded for very serious risk of bias (open-label designs enabling biased clinical decision-making, with large effects in Seitz 2023 [37] suggesting ascertainment bias rather than genuine treatment effects), serious inconsistency (null findings in the only blinded trial versus variable results in open-label studies), and very serious imprecision (total N = 148, with insufficient events for reliable estimation) [33,34,37,38]. The single adequately blinded trial (Rangon) found no benefit on the WHO Clinical Progression Scale (*p* > 0.3), ICU transfer (14% vs. 7%, *p* = 0.60), or mortality (0% vs. 7%, *p* = 1.0) [38].

For autonomic function (heart rate variability), evidence was rated very low based on a single trial (Corrêa, *n* = 52) that found no significant changes in any HRV parameter (SDNN, RMSSD, pNN50, LF, HF, LF/HF ratio) in either group over 14 days of treatment [7]. The evidence was downgraded for serious risk of bias and very serious imprecision.

For cognitive function, the certainty was rated very low based on two publications from a single trial [34,35]. The interim analysis (Uehara, *n* = 21) reported significant improvements in memory (*p* = 0.01) and attention (*p* = 0.04), but these findings were no longer statistically significant in the completed trial (Corrêa, *n* = 52). These false positive findings warranted downgrading for the very serious risk of bias, serious indirectness and very serious imprecision.

For safety and tolerability, the certainty was rated low, downgraded for serious risk of bias (differential adverse event attribution in open-label designs) and serious imprecision (N = 221 insufficient to detect rare adverse events; short treatment and follow-up durations precluding long-term safety assessment).

Although the JADAD scale, PEDro scale, and GRADE framework assessments were not pre-specified in the PROSPERO-registered protocol, they were incorporated during the conduct of the review as supplementary and complementary instruments to the primary Cochrane Risk of Bias 2 evaluation. This post-protocol addition was motivated by the recognition that multi-instrument methodological appraisal (trial-level quality assessment through both the JADAD and PEDro scales, and evidence-level certainty evaluation through GRADE) would afford a more comprehensive characterization of the overall strength of the evidence base. All three instruments are independently validated and widely employed in the systematic review literature, and their inclusion is considered a methodological enhancement that strengthens rather than compromises the integrity of the quality appraisal process. This update of the registered protocol is transparently acknowledged in accordance with best practice for systematic review conduct and reporting.

### 3.12. Outcomes

#### 3.12.1. Inflammatory Biomarkers

Four trials reported effects of VNS on inflammatory biomarkers with variable findings. The Rangon trial did not report inflammatory marker outcomes.

Tornero et al. (SAVIOR-I) reported significantly greater reductions in CRP at day 5 in the VNS group compared to standard of care (geometric mean ratio 0.62, 95% CI 0.42–0.92, *p* = 0.016), with an overall treatment effect across all timepoints (*p* = 0.011). On day 5, 87.5% of VNS patients had achieved normal CRP levels (<10 mg/L) compared to 50% of controls (*p* = 0.015). Procalcitonin also decreased significantly more in the VNS group at day 5 (*p* = 0.012). However, no significant between-group differences were observed for D-dimer, ferritin, or cytokine levels (TNF-α, IL-6, IL-1β, IFN-γ) at any time point.

Seitz et al. (2022) [36] reported within-group reductions in multiple inflammatory markers in VNS-treated patients: CRP decreased by 80% over 7 days (from mean 151.9 to 31.5 mg/dL), TNF-α decreased by 58% (from 19.3 to 8.1 pg/mL), D-dimer decreased by 66% (from 4.5 to 1.5 μg/mL), and IL-10 (anti-inflammatory) increased by 66% (from 2.7 to 7.0 pg/mL). Several between-group comparisons reached statistical significance: IL-6 and TNF-α changes from baseline to 4 h (both *p* = 0.048), D-dimer from baseline to 24 h (*p* = 0.025), CRP from baseline to 72 h (*p* = 0.003) and 168 h (*p* = 0.018), fibrinogen from baseline to 168 h (*p* = 0.002), and IL-10 from baseline to 72 and 168 h (*p* = 0.041 and *p* = 0.048).

Corrêa et al. (2022) [34] found that inflammatory markers decreased in both the VNS and control groups over time, reflecting the expected clinical course of COVID-19 with standard treatment. No significant between-group differences were observed for CRP, IL-6, or other inflammatory markers after appropriate consideration of multiple testing. Of mechanistic importance, this trial measured heart rate variability (HRV) as a direct physiological indicator of vagal activation and autonomic function [50,51]. The analysis revealed no significant change in any HRV parameter (time-domain or frequency-domain) in either group, and no between-group differences in HRV changes.

#### 3.12.2. Clinical Outcomes

Clinical outcomes were reported in five trials with inconsistent results. The only adequately blinded trial (Rangon et al.) found no significant improvement in the primary outcome (WHO 7-point Clinical Progression Scale score at Day 14 (*p* > 0.3)) or in secondary clinical outcomes, including ICU transfer rate (14% vs. 7% in VNS vs. sham, *p* = 0.60) or mortality (0% vs. 7%, *p* = 1.0). These null findings in the most methodologically rigorous trial are particularly informative.

Tornero et al. (SAVIOR-I) reported no significant differences in clinical respiratory outcomes, ICU admission rates, length of hospital stay, or mortality between VNS and control groups, despite the positive inflammatory marker findings. This discordance may indicate that the inflammatory marker findings were spurious, that the magnitude of inflammatory modulation was insufficient to translate into clinical improvement, or that the trial was underpowered for clinical outcomes.

Seitz et al. (2023) [37] reported dramatic differences in clinical outcomes: 0% intubation in VNS patients versus 50% in controls; 0% ECMO requirement versus 67% in controls; and 33% mortality versus 50% mortality. These effect sizes are implausibly large and unprecedented for any neuromodulation intervention. The absolute risk reductions of 50% for intubation and 67% for ECMO far exceed effects observed for proven COVID-19 therapies, including corticosteroids, which reduce mortality by approximately 8–10 percentage points, suggesting biased clinical decision-making in the context of open-label design.

#### 3.12.3. Cognitive Outcomes

Two publications from the Brazilian research group reported cognitive outcomes. Uehara et al. (2022, *n* = 21) [35] reported significant improvements in memory performance (*p* = 0.01) and attention (*p* = 0.04) in VNS patients compared to controls. However, in the larger Corrêa et al. (2022) [34] publication (*n* = 52) from the same trial, these cognitive findings were no longer statistically significant.

This pattern (where significant findings from a small interim analysis (*n* = 21) disappear when the complete sample is analyzed (*n* = 52)) represents a compelling empirical demonstration of false positive findings in small, underpowered studies. This observation has important implications for the interpretation of other small VNS trials reporting positive results.

#### 3.12.4. Safety and Tolerability

VNS was generally well-tolerated across all included trials with no serious adverse events attributed to the intervention. Seitz et al. (2022) [36] reported mean pain scores of 1.9 (range 0–3) on a 10-point visual analog scale during percutaneous auricular stimulation, with higher scores (mean 3.4, range 2–5) at the time of needle electrode placement. No patient discontinued treatment due to discomfort. Tornero et al. reported no serious VNS-related adverse events, though systematic recording of non-serious adverse events was limited by pandemic conditions. Rangon et al. noted that the auricular neuromodulation procedure was well-tolerated with no reported side effects. These safety findings are consistent with the established safety profile of non-invasive VNS from studies in other conditions [20,23].

## 4. Discussion

This systematic review identified six randomized controlled trials evaluating vagus nerve stimulation in 221 hospitalized patients with acute COVID-19. The central finding is that 83% of trials (5/6) were rated as high risk of bias according to the Cochrane RoB 2 framework, with the dominant sources of bias being inadequate blinding, substantial baseline imbalances, significant missing data, and extensive multiple testing without statistical correction. The single trial with adequate double-blinding and a credible sham control procedure (Rangon et al.) found null results across all assessed outcomes, including clinical progression, ICU transfer, and mortality. In contrast, the five high-risk-of-bias trials generally reported positive findings on various inflammatory markers and/or clinical outcomes.

Several additional observations from our analysis further undermine confidence in the positive findings. In Seitz et al. (2022) [36], markedly elevated baseline inflammatory markers in the VNS group create conditions strongly favoring regression to the mean (the statistical phenomenon whereby extreme values at baseline tend to move toward the population mean on subsequent measurement regardless of intervention [52]), thereby artificially inflating the appearance of treatment-related improvement. The near-universal absence of blinding across five of six trials compounds this problem considerably, as open-label designs are particularly problematic for outcomes influenced by clinical decision-making, including timing of ICU admission, intubation threshold, and discharge decisions, where knowledge of treatment assignment may systematically alter clinician behavior and assessment. This likely accounts for the implausibly large between-group differences in clinician-determined outcomes reported in Seitz et al. (2023) [37], where intubation rates were 0% in the VNS group versus 50% in controls and ECMO requirement was 0% versus 67%; differences in this magnitude are inconsistent with plausible biological effects of neuromodulation and more plausibly explained by biased clinical decision-making (whereby clinicians applied higher escalation thresholds in patients believed to be receiving active treatment) than by any true treatment effect. In Corrêa et al. (2022) [34], two independent concerns converge to undermine confidence in the reported findings: the co-intervention imbalance, whereby 100% of VNS patients but only 39% of controls received gastrointestinal drugs, introduces a confound that could directly influence inflammatory markers and clinical outcomes, rendering attribution of observed effects to VNS untenable; and the complete absence of heart rate variability changes despite claimed anti-inflammatory effects contradicts the proposed mechanism of action, suggesting either that effective vagal activation did not occur or that any observed inflammatory changes were not mediated through vagal pathways. Finally, the relationship between the Uehara [35] and Corrêa [34] publications provides direct empirical evidence of false positive findings failing to replicate: significant cognitive findings reported in the interim sample (memory performance *p* = 0.01, attention *p* = 0.04, *n* = 21) were no longer statistically significant upon completion of the larger sample (*n* = 52), a pattern that illustrates the unreliability of results from small, underpowered trials conducted without appropriate statistical correction and that should substantially temper confidence in other unreplicated positive findings across this literature.

The overall certainty of evidence for VNS in acute COVID-19 is very low for all efficacy outcomes and low for safety. According to the GRADE framework, very low certainty indicates that any estimate of effect is very uncertain, and the true effect is likely to be substantially different from the estimate of effect [43,44]. No factors warranting the upgrading of evidence certainty were identified. The magnitude of reported effects, where present, was inconsistent across trials and confined to high-risk-of-bias studies, precluding upgrading for a large effect. No dose–response relationship was observed across studies employing different stimulation parameters.

The supplementary assessment of trial quality using the JADAD scale and PEDro scale reinforced the findings of the primary Cochrane RoB 2 evaluation and GRADE assessment. JADAD scores were below the conventional threshold of adequate methodological quality in five of six included trials, with the single exception being the only double-blind sham-controlled trial in the evidence base. PEDro scores similarly fell below the accepted threshold for good methodological quality in five of six trials, with deficiencies concentrated in the domains of blinding, allocation concealment, and intention-to-treat analysis. The convergence of findings across independent quality appraisal instruments substantially strengthens the conclusion that the methodological limitations identified in this evidence base are not an artifact of any single assessment tool but reflect deficiencies in trial conduct that must be considered when interpreting the reported findings.

Our findings and conclusions differ substantially from those of Taha et al. (2024) [53], who conducted a meta-analysis of four RCTs (*n* = 180) and concluded that non-invasive VNS “may positively impact certain inflammatory markers” in COVID-19 patients. Several methodological differences between our systematic reviews may explain these discrepant conclusions. Risk of bias assessment methodology: Taha et al. rated the Tornero (SAVIOR-I) trial as “low” risk of bias, despite its open-label design without a sham control, 40–50% missing laboratory data, significant baseline age differences between groups, industry funding, and explicit acknowledgment of no multiplicity correction. Our application of the RoB 2 criteria, with attention to how each methodological feature could bias results, yielded a “high” risk of bias assessment for this trial across multiple domains. Similarly, Taha et al. rated 75% of the studies included as “low” risk or having only some concerns, whereas our assessment found 83% to be “high” risk of bias. This discrepancy appears to reflect the limited or superficial application of risk of bias criteria as a checklist rather than a critical evaluation of whether methodological features introduced systematic bias into the results. Study overlap identification: Taha et al. did not identify or address the apparent overlap between the Uehara and Corrêa publications, including both as independent studies in their analysis, despite sharing the same trial registry number (RBR-399t4g5), institutional affiliation, and overlapping authorship. This may have resulted in double-counting of patients in pooled analyses, artificially inflating precision and potentially biasing effect estimates. Our review identified this overlap and treated the publications as representing a single trial with interim (Uehara) and complete (Corrêa) data reporting. Exclusion of the only blinded trial: Most critically, Taha et al. excluded the Ragon trial from their meta-analysis, citing “different outcomes” as the rationale. However, Ragon was the only adequately blinded trial among all available studies and found null results across all outcomes. Excluding the most methodologically rigorous trial while including all inadequately blinded trials introduces systematic bias toward positive findings in the pooled estimate. This decision substantially undermines the validity of their meta-analytic conclusions. Mechanistic contradiction: Taha et al. did not address the mechanistic contradiction from Corrêa et al., whereby HRV (the direct measure of vagal activation) showed no change despite claimed anti-inflammatory effects. False positive demonstration: Taha et al. did not note the empirical demonstration of false positives whereby Uehara’s significant cognitive findings (*p* = 0.01, *p* = 0.04) disappeared in the larger Corrêa sample. This pattern directly illustrates the unreliability of small-study positive findings and should inform the interpretation of other positive results from similarly underpowered trials. These observations highlight a fundamental concern in evidence synthesis: quantitative meta-analysis of high-risk-of-bias studies produces “misleading precision around potentially biased estimates.” [30,31]. The mathematical precision of pooled effect estimates (narrow confidence intervals, small *p*-values) can create an illusion of robust evidence when the underlying studies are systematically biased in the same direction [54]. If all included studies are biased toward showing benefit (as occurs when all studies are open-label with expectation bias), pooling them produces a precise but biased estimate of effect. This would be a general methodological principle as expressed in the literature, and indeed the Cochrane Handbook explicitly cautions against pooling studies at “high” risk of bias, as this approach may compound rather than mitigate bias and can mislead clinical decision-making [31]. Our decision to pursue narrative synthesis rather than meta-analysis reflects adherence to this methodological principle.

The theoretical rationale for VNS in COVID-19 rests on activation of the cholinergic anti-inflammatory pathway (CAP) through vagal signaling to the spleen and other lymphoid organs, resulting in acetylcholine release and suppression of pro-inflammatory cytokine production via α7nAChR-mediated NF-κB inhibition. While this pathway has been convincingly demonstrated in rodent models of endotoxemia and sepsis [11,13], its clinical translation to humans remains uncertain. Recent comprehensive systematic reviews and meta-analyses have found no consistent evidence for anti-inflammatory effects of VNS in humans across various conditions and patient populations [26,27,28].

The mechanistic questions raised by the current evidence are substantial. The Corrêa trial’s finding of unchanged HRV despite claimed anti-inflammatory effects is particularly problematic. Heart rate variability, specifically the high-frequency (HF) component, reflects cardiac vagal modulation and serves as a validated non-invasive marker of vagal tone [55]. If VNS operates through vagal activation of the cholinergic anti-inflammatory pathway, increased vagal tone, reflected in increased high-frequency HRV power, should precede or accompany anti-inflammatory effects. However, HRV reflects cardiac parasympathetic modulation rather than splenic efferent activation directly and cannot therefore be considered a definitive measure of immunologically relevant vagal engagement [56]. The complete absence of HRV changes suggests one of several, speculative possibilities: (1) the stimulation parameters were insufficient to activate auricular vagal afferents; (2) auricular stimulation activates afferents but does not effectively engage the central circuits involved in the inflammatory reflex; (3) the cholinergic anti-inflammatory pathway is less robust in humans than in rodent models; or (4) any observed inflammatory changes are mediated through non-vagal mechanisms and are not true treatment effects.

Pathophysiological interpretation is further complicated by the heterogeneity of VNS approaches. Cervical stimulation targets both afferent and efferent vagal fibers, while auricular stimulation targets the purely afferent ABVN; whether this can engage the inflammatory reflex through central circuits remains unclear [16,57]. Stimulation parameters also varied substantially (frequency 1–25 Hz, variable intensity and duty cycles, duration from single sessions to 14+ days) without systematic optimization, and the optimal parameters for anti-inflammatory effects in humans remain unknown.

Our findings should be interpreted in the context of the broader literature on VNS for inflammatory conditions. Interest in VNS for COVID-19 continues, specifically for long-COVID sequelae, with ongoing and planned trials investigating various stimulation approaches [58,59]. However, enthusiasm for clinical translation should be tempered by recognition that the evidence base in humans remains limited. Schiweck et al. (2024) [26] conducted a comprehensive systematic review and meta-analysis examining the anti-inflammatory effects of VNS in humans across all conditions, concluding that there is no consistent evidence supporting anti-inflammatory efficacy. The authors noted substantial heterogeneity across studies, variable quality of evidence, and publication bias favoring positive findings. Similarly, de Melo et al. (2025) [27] found mixed results in their mechanistic analysis of VNS effects on inflammatory markers.

Although a previous systematic review and meta-analysis has examined VNS in COVID-19 [53], focusing primarily on inflammatory marker outcomes, we identified the need for a more comprehensive evaluation of the available evidence. The present review adopts a holistic approach, critically appraising not only inflammatory biomarker findings but also clinical outcomes (mortality, ICU admission, mechanical ventilation, length of stay), cognitive and functional outcomes, mechanistic data (heart rate variability as an indicator of vagal target engagement), and safety profiles across all included trials. Furthermore, we applied rigorous risk of bias assessment with detailed domain-level justifications, three independent quality appraisal instruments, identified potential study overlaps not previously recognized, and examined internal consistency between proposed mechanisms and reported outcomes. This comprehensive approach revealed critical methodological limitations and mechanistic contradictions that were not addressed in prior analyses, providing a more complete and nuanced understanding of the current evidence base. All of this, alongside the novel use of multiple AI tools for initial risk of bias assessment, followed by iterative synthesis and expert validation, represents the strengths of this endeavor.

Limitations include the small number of eligible trials and the substantial clinical and methodological heterogeneity that precluded quantitative synthesis. We were unable to obtain individual patient data for subgroup analyses or to definitively determine the extent of overlap between the Austrian and Brazilian study pairs. The included studies varied in VNS modality, stimulation parameters, patient populations, outcomes assessed, and follow-up duration, limiting the ability to draw definitive conclusions about specific approaches. We included only English-language publications, potentially missing relevant non-English studies. Publication bias cannot be excluded, as small negative trials may be less likely to reach publication than positive studies. A limitation of the present search strategy is the absence of EMBASE, attributable solely to the lack of institutional access at the time the searches were conducted. To compensate, several complementary strategies were systematically employed to maximize retrieval comprehensiveness: searches of multiple clinical trial registries (ClinicalTrials.gov, ICTRP), which represent the primary source of RCT registration independent of bibliographic database indexing; forward and backward citation tracking of all included studies and all cited materials; and manual screening of reference lists from relevant reviews. These measures substantially reduce the likelihood that eligible trials were overlooked, though the possibility of undetected EMBASE-indexed studies cannot be entirely excluded and is acknowledged as a residual limitation.

While the current evidence is insufficient to recommend VNS as adjunctive treatment for acute COVID-19, the therapeutic potential of this approach should not be dismissed. The pathophysiological pathway, targeting the cholinergic anti-inflammatory pathway, remains scientifically compelling, and signals of potential benefit on inflammatory parameters have emerged from several trials despite methodological limitations. These observations do not exclude the possibility that VNS may prove effective under rigorous evaluation conditions and support continued investigation rather than abandonment of the approach. Large-scale, double-blind, sham-controlled trials addressing the methodological shortcomings identified in this review are warranted to definitively establish whether VNS can fulfill its therapeutic promise.

Future research on VNS for COVID-19 or other inflammatory conditions should address the methodological limitations identified in this review. Essential design features for definitive trials include adequate statistical power based on realistic effect sizes informed by pilot data; sample sizes of 10–50 patients per arm are insufficient to detect clinically meaningful differences with acceptable precision. Double-blind design with credible sham control that mimics the sensory experience of active stimulation; for auricular approaches, sham stimulation of non-vagally innervated ear regions may be appropriate. Stratified randomization to ensure balance on key prognostic factors (age, disease severity, comorbidities). Pre-specified primary outcome with appropriate multiplicity correction for secondary outcomes; the primary outcome should be clinically meaningful (e.g., composite of mortality and need for mechanical ventilation). Mechanistic endpoints confirming target engagement, such as HRV changes demonstrating vagal activation. Complete outcome ascertainment with pre-specified approaches to missing data. Prospective protocol registration with transparent reporting per CONSORT guidelines.

The pandemic context that motivated rapid trial initiation may also have contributed to the methodological limitations observed; future trials conducted outside emergency conditions can and should apply more rigorous design standards.

## 5. Conclusions

This systematic review of six randomized controlled trials (*n* = 221 patients) evaluating vagus nerve stimulation in hospitalized COVID-19 patients found that the current evidence is insufficient to support VNS as an established therapeutic option, the evidence base being limited by methodological shortcomings. Five of six trials (83%) were rated high risk of bias according to the Cochrane RoB 2 framework; positive findings were confined to method-based weaker studies, while the only adequately blinded trial found null results across all outcomes. A mechanistic contradiction, absent HRV changes despite claimed anti-inflammatory effects, and an empirical false positive demonstration (significant cognitive findings that disappeared in a larger sample) further undermine confidence in reported benefits. A previous meta-analysis concluding potential benefit may reflect methodological differences, including risk of bias assessment stringency, the handling of potentially overlapping study populations, trial inclusion criteria, and the decision to pool studies despite substantial heterogeneity.

The biological rationale for targeting the cholinergic anti-inflammatory pathway in COVID-19 remains scientifically promising, and preliminary signals of potential anti-inflammatory effects have been observed across several trials. These findings, while insufficient to support clinical implementation, provide justification for continued investigation of this therapeutic approach. Definitive evaluation of VNS efficacy in COVID-19 will require large-scale, multicenter, double-blind, sham-controlled trials with adequate statistical power, pre-specified primary outcomes, appropriate multiplicity adjustment, and incorporation of biomarkers confirming vagal target engagement.

## Figures and Tables

**Table 1:** Characteristics of Included Randomized Controlled Trials.

Study	N	VNS Type/Device	Control	Blinding	Duration	Country	Primary Outcomes
Tornero 2022 (SAVIOR-I) [33]	97	tcVNS (gammaCore Sapphire)	Standard of care	Open-label	Through discharge (≤15 days)	Spain	CRP, inflammatory markers, clinical outcomes
Rangon 2021 (SOS COVID-19) [38]	29	Auricular (semi-permanent needles)	Sham (pressure, no needles)	Double-blind	Single application	France	WHO Clinical Progression Scale Day 14
Seitz 2022 [36]	10	paVNS (AuriStim)	Standard of care	Open-label	Mean 12 days	Austria	Inflammatory markers (IL-6, TNF-α, CRP, IL-10)
Seitz 2023 [37]	12	paVNS (AuriStim)	Standard of care	Open-label	ICU stay	Austria	Clinical outcomes (intubation, ECMO, mortality)
Corrêa 2022 [34]	52	taVNS	Standard of care	Open-label	14 days	Brazil	Inflammatory markers, HRV, and cognition
Uehara 2022 * [35]	21	taVNS	Standard of care	Open-label	14 days	Brazil	Cognition, inflammatory markers

* Uehara 2022 [35] shares trial registration (RBR-399t4g5) with Corrêa 2022 [34]; it appears to represent an interim analysis from the same trial. CRP, C-reactive protein; ECMO, extracorporeal membrane oxygenation; HRV, heart rate variability; IL, interleukin; paVNS, percutaneous auricular vagus nerve stimulation; taVNS, transcutaneous auricular vagus nerve stimulation; tcVNS, transcutaneous cervical vagus nerve stimulation; TNF-α, tumor necrosis factor-alpha; WHO, World Health Organization.

**Table 2:** Risk of Bias Summary by Domain (RoB 2 Tool).

Study	D1: Randomization	D2: Deviations	D3: Missing Data	D4: Measurement	D5: Reporting	Overall
Tornero 2022 [33]	Some concerns	HIGH	HIGH	Low	HIGH	HIGH
Rangon 2021 [38]	Some concerns	Low	Low	Low	Some concerns	Some concerns
Seitz 2022 [36]	HIGH	HIGH	Low	Low	HIGH	HIGH
Seitz 2023 [37]	Some concerns	HIGH	Low	HIGH	HIGH	HIGH
Corrêa 2022 [34]	Some concerns	HIGH	Some concerns	Low	Some concerns	HIGH
Uehara 2022 [35]	Some concerns	HIGH	Some concerns	HIGH	HIGH	HIGH

D1, Domain 1: Bias arising from the randomization process; D2, Domain 2: Bias due to deviations from intended interventions; D3, Domain 3: Bias due to missing outcome data; D4, Domain 4: Bias in measurement of the outcome; D5, Domain 5: Bias in selection of the reported result. “HIGH” indicates high risk of bias. “Low” indicates low risk of bias. “Some concerns” indicates methodological uncertainty, typically arising from incomplete reporting or minor limitations that may introduce potential, but not definitive, bias.

**Table 3:** Detailed Risk of Bias Judgments with Supporting Rationales.

Study	Domain: Judgment	Supporting Rationale
Tornero 2022 [33]	D1: Some concerns	Computer-based 1:1 randomization is described, but allocation concealment is not detailed. Significant baseline differences: mean age 55.5 vs. 61.3 years (*p* = 0.022); more severe cases in the VNS group (15% vs. 4%). Baseline imbalances were not adjusted for in the analysis.
	D2: HIGH	Open-label design with no sham control. The GammaCore device produces perceptible sensory stimulation (tingling, muscle contraction). Clinical decisions (ICU admission, escalation, discharge) made by unblinded clinicians aware of treatment assignment.
	D3: HIGH	13/110 randomized patients (12%) were excluded for missing baseline data. Laboratory values are missing for 40–50% of patients at various timepoints. The authors acknowledged data collection challenges. No imputation, no sensitivity analyses for missing data.
	D5: HIGH	Multiple inflammatory markers and clinical outcomes were tested without multiplicity correction. The authors stated ‘no adjustments for multiple comparisons.’ Industry funding from electroCore Inc. introduces a potential conflict of interest.
Rangon 2021 [38]	D1: Some concerns	Computer-based block randomization is described with adequate concealment. Notable baseline imbalances despite randomization: median age 75.5 vs. 65 years; 79% vs. 47% male; these known prognostic factors were not adjusted for.
	D2: Low	Double-blind design with credible sham procedure (pressure with an empty applicator). Opaque bandages covered both ears identically. Best blinding among all included trials. Successful blinding was not formally assessed but appears adequate.
	D5: Some concerns	Multiple outcomes assessed (WHO scale, ICU transfer, mortality, inflammatory markers) without clear hierarchy or multiplicity adjustment. Study stopped early after interim analysis due to recruitment difficulties; pre-specified sample size not achieved.
Seitz 2022 [36]	D1: HIGH	Massive baseline imbalances in key outcome variables: TNF-α 3-fold higher in VNS (19.3 vs. 6.3 pg/mL); IL-6 2.6-fold higher (341 vs. 129 pg/mL); CRP 1.8-fold higher (152 vs. 84 mg/dL). Creates a strong regression to the mean artifact, favoring the apparent VNS effect.
	D2: HIGH	Open-label design. Percutaneous needles inserted into the auricle produce an obvious sensation. No sham control. No blinding of patients, clinicians, or outcome assessors.
	D5: HIGH	Eight inflammatory markers measured at up to 20 timepoints with between-group comparisons at each; no multiplicity correction. Possible patient overlaps with Seitz 2023, with different outcomes selectively reported; the relationship between publications is unclear.
Seitz 2023 [37]	D2: HIGH	Open-label design with no sham. Clinical decisions (intubation timing, ECMO initiation) made by unblinded ICU clinicians. Knowledge of the VNS assignment may have raised the threshold for escalation in the treatment group.
	D4: HIGH	Implausibly large effects for clinician-determined outcomes (0% vs. 67% ECMO; 0% vs. 50% intubation). Effect sizes are inconsistent with any known VNS mechanism and unprecedented in neuromodulation literature. The pattern suggests bias rather than treatment effect.
	D5: HIGH	Reports clinical outcomes from the cohort that may overlap with Seitz 2022 (inflammatory markers). The pattern suggests selective outcome reporting across multiple publications from the same dataset without transparency.
Corrêa 2022 [34]	D1: Some concerns	Randomization method described. Baseline IL-6 34% higher, and CRP 22% higher in the control group; the direction of imbalance may underestimate rather than inflate the treatment effect, but this was not adjusted for in the analysis.
	D2: HIGH	Open-label with no sham. Critical co-intervention imbalance: 100% of VNS patients received GI drugs vs. only 39% of controls (*p* = 0.001). This systematic difference was unexplained and unadjusted—could independently affect inflammatory and clinical outcomes.
	D3: Some concerns	Differential HRV data exclusion: 7.7% VNS vs. 19.2% control excluded from autonomic analysis. No investigation of whether excluded participants differed systematically; potential for selection bias in HRV findings.
Uehara 2022 [35]	D2: HIGH	Open-label design with no sham control. Cognitive assessments performed by unblinded examiners. Expectation bias likely influenced the administration, scoring, and interpretation of neuropsychological tests.
	D4: HIGH	Cognitive outcomes (memory, attention, executive function) are subjective measures requiring examiner judgment. Unblinded assessment introduces high susceptibility to expectation and ascertainment bias.
	D5: HIGH	Shares trial registration (RBR-399t4g5) with Corrêa 2022; represents interim analysis published separately. Significant cognitive findings (*p* = 0.01, 0.04) did not replicate in the larger Corrêa sample—empirical demonstration of false positives from underpowered interim analysis.

**Table 4:** JADAD Scale Assessment.

Study	Q1 Randomized?	Q2 Method Appropriate?	Q3 Double-Blind?	Q4 Blinding Method Appropriate?	Q5 Withdrawals Described?	Total Score (0–5)
Tornero et al., 2022 (SAVIOR-I) [33]	Y	Y	N	N	Y	3/5
Rangon et al., 2021 (SOS COVID-19) [38]	Y	Y	Y	Y	Y	5/5
Seitz et al., 2022 [36]	Y	N	N	N	N	1/5
Seitz et al., 2023 [37]	Y	Y	N	N	N	2/5
Corrêa et al., 2022 [34]	Y	Y	N	N	Y	3/5
Uehara et al., 2022 [35]	Y	Y	N	N	N	2/5

JADAD Scale Scores for Included RCTs (N = 6). Y = criterion satisfied; N = criterion not satisfied. Score interpretation: ≥3 = adequate methodological quality; <3 = inadequate quality.

**Table 5:** PEDro Scale Assessment.

Study	2 Random Alloc.	3 Concealed Alloc.	4 Baseline Compar.	5 Blind Subjects	6 Blind Therapists	7 Blind Assessors	8 >85% Follow-up	9 ITT Analysis	10 Between- Group Stats	11 Point Measures	Total (0–10)
Tornero et al., 2022 [33]	Y	N	N	N	N	N	Y	N	Y	Y	5/10
Rangon et al., 2021 [38]	Y	Y	Y	Y	N	Y	Y	Y	Y	Y	9/10
Seitz et al., 2022 [36]	Y	N	N	N	N	N	Y	N	Y	Y	4/10
Seitz et al., 2023 [37]	Y	N	N	N	N	N	Y	N	Y	Y	4/10
Corrêa et al., 2022 [34]	Y	N	Y	N	N	N	N	N	Y	Y	4/10
Uehara et al., 2022 [35]	Y	N	N	N	N	N	Y	N	Y	Y	4/10

PEDro Scale Scores for Included RCTs (N = 6). Y = criterion satisfied; N = criterion not satisfied. Items 2–11 scored. Score interpretation: ≥6 = good methodological quality; 4–5 = fair; <4 = poor. ITT = intention-to-treat analysis.

**Table 6:** GRADE Evidence Profile.

Outcome	No. of Studies (N)	Risk of Bias	Inconsistency	Indirectness	Imprecision	Publication Bias	Overall Certainty	Importance
Inflammatory biomarkers (CRP, IL-6, TNF-α)	4 (171)	Very serious	Very serious	Not serious	Very serious	Suspected	VERY LOW	CRITICAL
Clinical outcomes (mortality, ICU, ventilation)	4 (148)	Very serious	Serious	Not serious	Very serious	Suspected	VERY LOW	CRITICAL
Autonomic function (heart rate variability)	1 (52)	Serious	Not applicable	Not serious	Very serious	Undetected	VERY LOW	IMPORTANT
Cognitive function	2 (73)	Very serious	Not applicable	Serious	Very serious	Suspected	VERY LOW	IMPORTANT
Safety and tolerability	6 (221)	Serious	Not serious	Not serious	Serious	Undetected	LOW	CRITICAL

GRADE certainty ratings: HIGH = further research is very unlikely to change confidence in the effect estimate; MODERATE = further research is likely to have an important impact; LOW = further research is very likely to have an important impact; VERY LOW = any estimate of effect is very uncertain. Color coding: green = no downgrading; yellow = downgraded one level; red = downgraded two levels.

**Table 7:** GRADE Summary of Findings.

Outcome	No. of Participants (Studies)	Effect with VNS	Effect with Control	Relative Effect (95% CI)	Certainty of Evidence (GRADE)
CRP reduction	171 (4 RCTs)	Variable reduction reported in open-label trials	Natural decline with standard care	GMR 0.62 (0.42–0.92) in Tornero only; null in Corrêa	VERY LOW
IL-6/TNF-α	62 (2 RCTs)	Nominal reductions in Seitz (*n* = 10); null in Corrêa (*n* = 52)	Parallel reductions in controls	No consistent between-group difference	VERY LOW
Mortality	148 (4 RCTs)	0–33% across trials	7–50% across trials	Non-significant; 0% vs. 7% in blinded trial (Rangon)	VERY LOW
ICU admission/ventilation/ECMO	148 (4 RCTs)	0–50% intubation; 0% ECMO in VNS (Seitz 2023 [37])	50–67% intubation; 67% ECMO in controls (Seitz 2023 [37])	Implausibly large differences in open-label; null in blinded trial	VERY LOW
Heart rate variability	52 (1 RCT)	No significant change in any HRV parameter	No significant change	No between-group differences (SDNN, RMSSD, HF, LF)	VERY LOW
Cognitive function	73 (2 RCTs)	Significant in interim (*n* = 21); null in completed trial (*n* = 52)	Not assessed in a blinded comparison	False positive demonstrated: *p* = 0.01 disappeared in a larger sample	VERY LOW
Adverse events	221 (6 RCTs)	No serious adverse events; pain 1.9/10	Not applicable	Well-tolerated; no discontinuations for discomfort	LOW

GMR, geometric mean ratio; CI, confidence interval; CRP, C-reactive protein; IL-6, interleukin-6; TNF-α, tumor necrosis factor-alpha; ICU, intensive care unit; ECMO, extracorporeal membrane oxygenation; HRV, heart rate variability; SDNN, standard deviation of NN intervals; RMSSD, root mean square of successive differences; HF, high frequency; LF, low frequency. GRADE certainty ratings apply to the body of evidence for each outcome.

## Data Availability

No new data were created or analyzed in this study.

## Supplementary Materials

The following supporting information can be downloaded at: https://www.mdpi.com/article/10.3390/medicina62040649/s1, PRISMA 2020 Checklist: Vagus Nerve Stimulation for COVID-19 hospitalized patients: A Systematic Review and Critical Appraisal of Randomized Controlled Trials. Reference [29] is cited in the Supplementary Materials.
